# Effects of Artificially Reproduced Fluctuations in Sunlight Spectral Distribution on the Net Photosynthetic Rate of Cucumber Leaves

**DOI:** 10.3389/fpls.2021.675810

**Published:** 2021-06-15

**Authors:** Ryo Matsuda, Hiroki Ito, Kazuhiro Fujiwara

**Affiliations:** Department of Biological and Environmental Engineering, Graduate School of Agricultural and Life Sciences, The University of Tokyo, Tokyo, Japan

**Keywords:** fluctuating light, light-emitting diode, light quality, LED artificial sunlight source system, photosynthetic photon flux density, spectral photon flux density distribution

## Abstract

The effects of photosynthetic photon flux density (PPFD) fluctuations in sunlight have already been investigated; however, the spectral photon flux density distribution (SPD) has hardly been considered. Here, sunlight SPD fluctuations recorded for 200 min in October in Tokyo, Japan were artificially reproduced using an LED-artificial sunlight source system. The net photosynthetic rate (*P*_n_) of cucumber leaves under reproduced sunlight was measured and compared with the *P*_n_ estimated from a steady-state PPFD–*P*_n_ curve for the same leaves. The measured and estimated *P*_n_ agreed except when the PPFD was low, where the measured *P*_n_ was lower than the estimated *P*_n_. The ratio of measured *P*_n_ to estimated *P*_n_ was 0.94–0.95 for PPFD ranges of 300–700 μmol m^–2^ s^–1^, while the value was 0.98–0.99 for 900–1,300 μmol m^–2^ s^–1^, and the overall ratio was 0.97. This 3% reduction in the measured *P*_n_ compared with the *P*_n_ estimated from a steady-state PPFD–*P*_n_ curve was significantly smaller than the approximately 20–30% reduction reported in previous experimental and simulation studies. This result suggests that the loss of integral net photosynthetic gain under fluctuating sunlight can vary among days with different fluctuation patterns or may be non-significant when fluctuations in both PPFD and relative SPD of sunlight are taken into consideration.

## Introduction

The spectral photon-flux-density distribution (SPD) is a distribution of photon flux density (PFD) per unit wavelength within a defined wavelength range. The SPD can be characterized by two aspects: the integral of spectral PFD and the relative SPD. As an index of the former factor, the photosynthetic PFD (PPFD), with an amount of PFD between 400 and 700 nm, is often used. The latter factor is the “shape” of the SPD curve and may sometimes be called light quality. As elements of the light environment, both PPFD (Boardman, 1977; Björkman, 1981) and relative SPD (McCree, 1972; Inada, 1976) significantly affect the net photosynthetic rate (*P*_n_) of leaves.

The SPD of sunlight in open fields and greenhouses fluctuates during the daytime at various time scales, from seconds to hours, because of a change in solar altitude, clouds covering the sun, leaf movement due to wind, and so on. Recently, the effects of PPFD fluctuations on instantaneous leaf photosynthesis have been intensively studied (for reviews, see Kaiser et al., 2015, 2018; Yamori, 2016; Vialet-Chabrand et al., 2017a; Murchie et al., 2018; Slattery et al., 2018; Tanaka et al., 2019). Reportedly, photosynthetic performance under fluctuating PPFD conditions is different from that under constant PPFD conditions. Most previous studies employed simple periodic fluctuations in PPFD in which PPFD alternated between two PPFD levels (Leakey et al., 2002; Kono et al., 2014, 2017, 2020; Sejima et al., 2014; Kono and Terashima, 2016; Yamori et al., 2016; Yang et al., 2019; Bhuiyan and van Iersel, 2021) or a single event involving an increase or decrease in PPFD (Kaiser et al., 2016; Qu et al., 2016; Soleh et al., 2017; Zhang et al., 2019). Although these studies demonstrated the significance of physiological responses to fluctuating light, the PPFD fluctuation patterns differ from complex fluctuation patterns observed in open fields and greenhouses under sunlight.

Vialet-Chabrand et al. (2017b) reproduced a sunlight PPFD fluctuation measured on a relatively clear day using a light-emitting diode (LED) light source. The researchers measured a diurnal change in leaf *P*_n_ in *Arabidopsis thaliana* under the conditions where PPFD fluctuated below 1,500 μmol m^–2^ s^–1^ and compared it with the *P*_n_ predicted from the separately determined PPFD-response curve of steady-state *P*_n_. They reported that the measured *P*_n_ tended to be lower than the predicted *P*_n_ and that the difference between the measured and predicted *P*_n_ integrated over the diurnal period was 19–30%. Similarly, model simulation studies reported that the daily integral net photosynthetic gain under sunlight where PPFD fluctuated was calculated as 21% lower than that estimated by assuming that steady-state photosynthesis was attained at any moment (Taylor and Long, 2017; Tanaka et al., 2019). The reduction in *P*_n_ by PPFD fluctuations was thought to be mainly attributed to the delayed response of photosynthesis to an increase in PPFD, i.e., photosynthetic induction. Photosynthetic induction comprises three processes: (i) the induction of photosynthetic electron transport reactions in the thylakoid membrane, (ii) the activities of Calvin cycle enzymes including ribulose-1,5-bisphosphate carboxylase/oxygenase (Rubisco), and (iii) gas diffusion conductance including stomatal opening, each has a different time to respond of approximately 1–2 min, 5–10 min, and 10–30 min, respectively (Pearcy, 1990; Tanaka et al., 2019; Kimura et al., 2020; Yamori et al., 2020). It has been considered increasingly important to understand the nature of photosynthesis under sunlight with fluctuating PPFD and its underlying physiological mechanisms for genetic improvements of related traits (e.g., Adachi et al., 2019; Kimura et al., 2020; Yamori et al., 2020). In addition, fluctuations in environmental factors other than PPFD (e.g., CO_2_ concentration, air temperature, relative humidity) have also been discussed (Kaiser et al., 2015; Yamori, 2016). On the other hand, most of the current greenhouse crop growth models (e.g., TOMSIM, Heuvelink, 1995, 1999) calculate leaf *P*_n_ in changing environments using parameters obtained with the assumption of steady-state conditions. However, such models simulate crop growth reasonably well under a wide range of growth conditions (e.g., Heuvelink, 1999; Heuvelink and Dorais, 2005; Heuvelink et al., 2008), indirectly suggesting that steady-state photosynthetic parameters are not too inappropriate to simulate leaf *P*_n_ of greenhouse crops under sunlight. Furthermore, a recent simulation study stated that the daily integral net photosynthetic gain calculated considering the delayed response of photosynthesis to an increased PPFD under various patterns of diurnal sunlight PPFD fluctuation was, on average, only 3–6% lower than *P*_n_ calculated assuming a steady-state (Murakami and Jishi, 2021). Thus, further verification is needed as to whether the approximately 20–30% reduction in *P*_n_ is a typical value under various fluctuating light conditions.

In contrast to PPFD reproduction, relative SPD or “light quality,” the other important aspect of sunlight SPD, has hardly been considered. For example, the light sources used in previous studies to artificially reproduce sunlight PPFD fluctuations were a commercial LED light source (Vialet-Chabrand et al., 2017b) and an LED light source attached to a commercial portable photosynthesis system (Adachi et al., 2019; Kimura et al., 2020; Yamori et al., 2020), of which the relative SPDs were completely different from those of sunlight. It is known that factors characterizing relative SPD, such as the proportions of blue, red, and far-red light and/or their ratios, are known to significantly influence instantaneous photosynthesis (e.g., Hogewoning et al., 2010; Murakami et al., 2016). Furthermore, Kono et al. (2017, 2020) clarified the importance of far-red light in the photosynthetic response to fluctuating PPFD; periodic PPFD fluctuations without far-red light caused photoinhibition of photosystem II, while it was suppressed when far-red light was added. Thus, it is strongly desired that not only PPFD but also the relative SPD of sunlight be reproduced when we evaluate the effects of sunlight fluctuation on photosynthesis and intend to extrapolate the results to open field or greenhouse crop production. On the other hand, investigating photosynthesis under sunlight in an actual open field or a greenhouse may be another option to elucidate the responses of photosynthesis to fluctuating light. However, such field experiments do not allow us to confirm the reproducibility of the results obtained. To ensure reproducibility, laboratory experiments under a controlled environment must be useful.

Fujiwara and Sawada (2006); Fujiwara et al. (2007), and Fujiwara and Yano (2011) have been developing an LED-artificial sunlight source (LASS) system. A second-generation LASS system (Fujiwara et al., 2013) can produce SPDs at the same level as full irradiation of ground-level sunlight, within a range of 380–940 nm, with a high approximation accuracy at the light outlet of 7.1 cm^2^ (30 mm*ϕ*). Moreover, it also has a time-varying (dynamic) light production program and can change the SPD at the light outlet to an arbitrarily modified SPD at an arbitrarily set time interval of more than 2 s. To our knowledge, this system is the most appropriate for elucidating the effects of sunlight SPD fluctuations, taking both PPFD and relative SPD into consideration, as well as ensuring a high reproducibility of sunlight SPD fluctuations.

In this study, we measured sunlight SPD fluctuations and artificially reproduced them using the LASS system. Characteristics of the measured sunlight SPD fluctuations and reproducibility of PPFD and relative SPD with the LASS system were evaluated. Then, the *P*_n_ of cucumber leaves under reproduced sunlight was compared with the *P*_n_ estimated from a steady-state PPFD–*P*_n_ curve of the same leaves.

## Materials and Methods

### Measurement of Fluctuations in Sunlight SPD

Fluctuations in sunlight SPD were measured at the top of a seven-storied building located in Bunkyo, Tokyo, Japan (35°43′N) with a spectroradiometer (MS-720, EKO Instruments Co., Ltd., Tokyo, Japan). The SPDs between 350 and 1,050 nm were measured and recorded once every 15 s. To protect the spectroradiometer from sudden strong wind and rain, it was placed in a box (450 mm × 450 mm × 300 mm) covered with a fluoropolymer film (F-CLEAN Clear, AGC Green-Tech Co., Ltd., Tokyo, Japan) with an almost constant spectral transmittance (>90%) within the wavelength range measured. Measurements were repeated several times from April to October 2017. Data collected from 11:10 to 14:30 on October 12, 2017, in which relatively large amplitudes and frequent fluctuations in PPFD were observed, were selected for reproduction. The measurement periods of 200 min (3 h and 20 min) corresponded to the maximum number of storable data (800) of the spectroradiometer.

### Reproduction of Fluctuations in Sunlight SPD With an LED-Artificial Sunlight Source System

**FIGURE 1:**
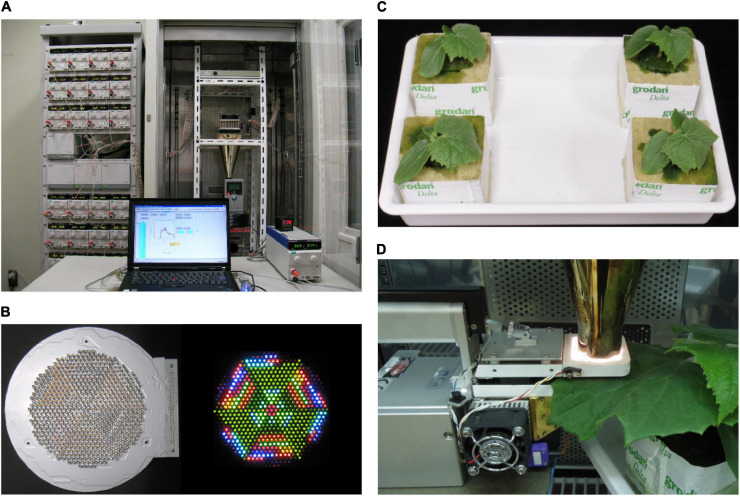
**(A)** The LED-artificial sunlight source (LASS) system. Left: 32 DC power supplies, a DC power supply controller, and three controller expansion units in a rack; right: the light source unit comprising an LED module, a cooling unit of the LED temperature control system, and a hollow conical reflection condenser, and a spectroradiometer in a temperature-controlled chamber; bottom: a DC power supply and a PID controller of the LED temperature control system and a laptop computer. **(B)** Bottom views of the LED module when all LEDs are off (left) and on (right). **(C)** 13-day-old cucumber seedlings grown under phosphor-converted white LED light. **(D)** During the measurement of net photosynthetic rate (*P*_n_), a part of a cucumber leaf was sandwiched in a leaf chamber of the portable photosynthesis system, and the surface of transparent film covering the top of the leaf chamber was placed in contact with the light outlet of the hollow conical reflection condenser of the LASS system.

Hardware and software system configurations of the second-generation LASS system were described in detail in Fujiwara et al. (2013). The hardware system comprises a light source unit, an LED temperature control system, and an SPD control system (Figure 1A). The light source unit comprises an LED module containing 625 LEDs with 32 different peak wavelengths (385–910 nm) (Figure 1B) and a hollow conical reflection condenser that condenses and mixes light from the LEDs to the light outlet of 7.1 cm^2^. The SPD control system comprises 32 direct current (DC) power supplies, a DC power supply controller, controller expansion units, and a laptop computer used to send voltage value signals to the DC power supply controller. The software installed in the computer enables production of the desired SPD at the light outlet by transmitting a set of appropriate, previously determined voltage signals to the DC power supply controller, which is then applied to each type of LED in the light source unit. According to the original procedure (Fujiwara et al., 2013), four-step procedures are taken to determine the set of appropriate voltages: (i) preparation of a voltage–spectral irradiance database; (ii) calculation of the set of appropriate voltages; (iii) re-approximation using feedback control; and (iv) light production. In this study, we did not use the re-approximation function. This function can minimize the difference in spectral distributions between the reproduced light and target light using feedback control with a spectroradiometer (Fujiwara et al., 2013). However, roughly 10 min was needed as one routine operation for each of the SPDs that we wanted to reproduce. In this study, we had 800 SPD data points to reproduce, and too much time was needed to finish the procedure; thus, we had to omit the third step for the use of the re-approximation function.

### Plant Materials and Growth Conditions

**FIGURE 2:**
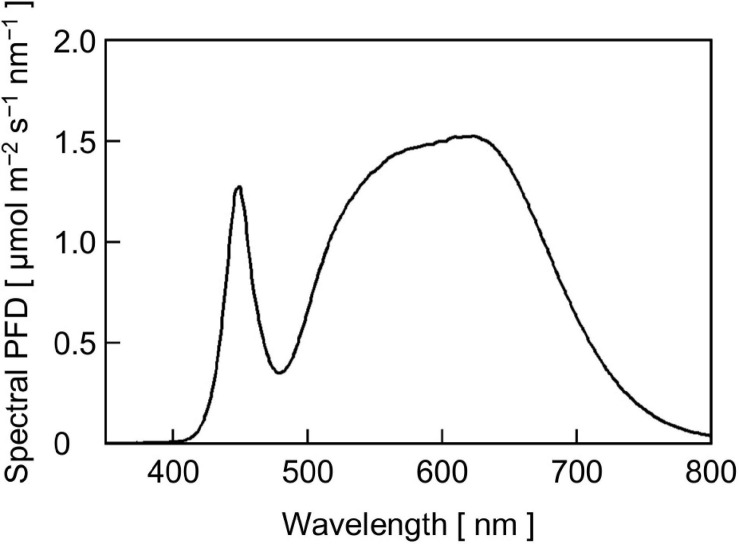
The SPD of phosphor-converted white LED light for cucumber seedling growth at a PPFD of 300 μmol m^–2^ s^–1^.

Cucumber (*Cucumis sativus* L. ‘Hokushin’, Takii & Co., Ltd., Kyoto, Japan) seeds were sown into moistened rockwool cubes (AO36/40, ROCKWOOL B.V., Roermond, the Netherlands) in a plug tray. Then, the tray was placed in a temperature-controlled growth chamber (MIR-554-PJ, PHC Holdings Corp., Tokyo, Japan) equipped with an LED panel [HMW120DC6 (1N-40Y), Kyoritsu Densho Co., Ltd., Osaka, Japan] composed of phosphor-converted white LEDs (GSPW1651NSE-40Y-TR, Stanley Electric Co., Ltd., Tokyo, Japan) (Figure 2). The seedlings were grown at a PPFD of 300 μmol m^–2^ s^–1^ at the tops of plants for 16 h d^–1^ and air temperatures of 25/20°C (day/night). The growth chamber was ventilated with external air using an air pump with the number of air exchanges of 1.0 h^–1^. At 7 days post-seeding, seedlings were transplanted onto rockwool cubes (Delta 6.5G, ROCKWOOL B.V.) and grown for another week under the same environmental conditions. The rockwool cubes were subirrigated once per day or every 2 days with a nutrient solution (prescription A, OAT Agrio Co., Ltd., Tokyo, Japan) at an electrical conductivity of 0.13 S m^–1^.

### Measurement of Leaf Gas Exchange Rates

The gas exchange rates of the first true leaves of the 13- to 15-day-old cucumber seedlings (Figure 1C) were measured using a portable photosynthesis system (LI-6400XT, LI-COR, Inc., Lincoln, United Kingdom). A leaf chamber of the portable photosynthesis system was not equipped with any light source provided by the manufacturer. The light outlet of the hollow conical reflection condenser of the LASS system was placed in contact with a surface of 2 × 3-cm transparent polypropylene film covering the leaf chamber (Figure 1D). Environmental conditions of the leaf chamber, other than PPFD, were set as follows: CO_2_ concentration of incoming air was 420 μmol mol^–1^, air temperature was 25°C, and relative humidity was 70%. The airflow rate to the leaf chamber was 500 μmol s^–1^. Measurements consisted of (1) changes in gas exchange rates under the reproduced sunlight and (2) steady-state *P*_n_ in response to PPFD. For (1), leaves were first kept at a constant PPFD of 500 μmol m^–2^ s^–1^ with a reference sunlight spectrum, which is defined by IEC 60904-3:2019 (International Electrotechnical Commission, 2019), for 20 min. The reference sunlight spectrum is defined for the global (direct and diffuse) solar radiation and at an air mass of 1.5. Leaves were then irradiated with light with an SPD at the beginning (0 min) of the reproduced sunlight (PPFD ca. 1,200 μmol m^–2^ s^–1^) for 20 min. Subsequently, leaves were irradiated with the reproduced sunlight for 200 min. The SPD was changed every 15 s. *P*_n_ and stomatal conductance (*g*_s_) were recorded every 3 s, and five gas exchange measurement data (3, 6, 9, 12, and 15 s) were recorded for each SPD of light. The means of the gas exchange parameters collected at 12 and 15 s were regarded as corresponding to the SPD of light to minimize the effects of the transient responses of the LASS system and the portable photosynthesis system. The readings of the reference and sample infrared gas analyzers (IRGAs) were matched after the sample gas was temporarily passed through the reference IRGA once every 20 min. For (2), leaves were first kept at a constant PPFD of 400 μmol m^–2^ s^–1^ with a relative SPD of the reference sunlight for 20 min. Then, leaves were irradiated with light with a relative SPD of the reference sunlight at different PPFD levels in the following order: 1,200, 1,000, 800, 600, 400, 200, and 0 μmol m^–2^ s^–1^. Each PPFD level was maintained for 20 min, and the mean *P*_n_ and *g*_s_ values for the last 5 min (15–20 min) were regarded as the steady-state values. Matching of the reference and sample IRGAs was carried out at 14–15 min after each PPFD level was attained.

We used 12 plants for measurements. Six plants were first subjected to measurement (1) followed by measurement (2), while the other six were subjected to measurements in the opposite order. Because no significant differences were found in the results between the two irradiation patterns, data for 12 plants were averaged irrespective of the irradiation pattern order.

The steady-state *P*_n_ averaged for 12 plants in response to PPFD was fitted with the following nonrectangular hyperbolic function (Johnson and Thornley, 1984) using the least-squares method: *P*_n_ = {*ϕI* + *P*_max_ − [(*ϕI* + *P*_max_) − 4*θϕIP*_max_]^0.5^} / 2*θ* − *R*_d_, where *I* is PPFD, mol m^–2^ s^–1^; *ϕ* is the initial slope, mol mol^–1^; *P*_max_ is the maximum rate of gross photosynthetic rate, mol m^–2^ s^–1^; *θ* is the convexity of the curve, dimensionless; and *R*_d_ is the dark respiratory rate, mol m^–2^ s^–1^.

## Results and Discussion

### Time Course of Sunlight SPD

**FIGURE 3:**
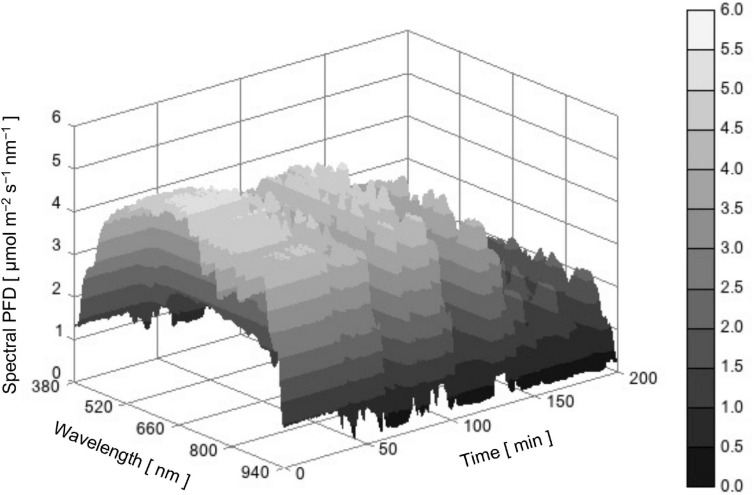
Time course of sunlight SPD between 380 and 940 nm measured in Bunkyo, Tokyo, Japan (35°43’N) from 11:10 to 14:30 on October 12, 2017.

**FIGURE 4:**
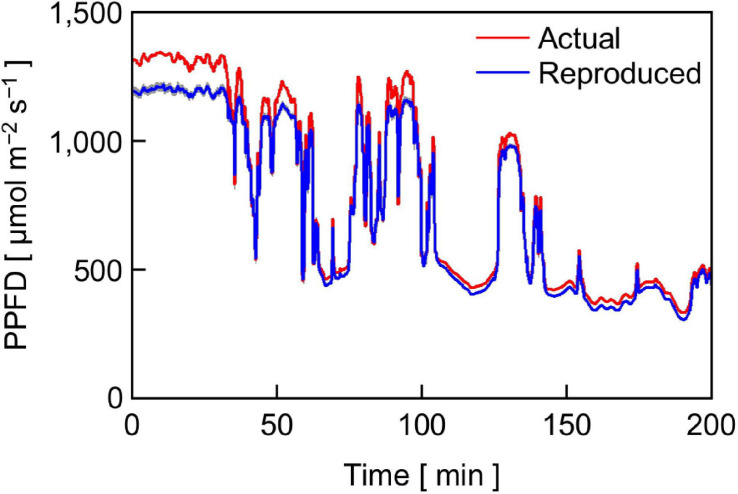
Time course of PPFD of actual sunlight measured in Bunkyo, Tokyo, Japan (35°43’N) from 11:10 to 14:30 on October 12, 2017 and that of sunlight reproduced with the LASS system. The height of gray area at a given time represents the standard deviation of the PPFD of the reproduced sunlight (*n* = 12).

Figure 3 is a three-dimensional surface plot showing the time course of SPD of actual sunlight between 380 and 940 nm. When focusing on PPFD (red line in Figure 4), the value was approximately 1,300 μmol m^–2^ s^–1^ at the beginning of measurement and then fluctuated in the range between 1,300 and 400 μmol m^–2^ s^–1^ because clouds sometimes covered the sun and direct solar radiation was largely attenuated. The relative SPD, or the shape of the SPD, may not be apparently different among times (Figure 3). However, sunlight with a lower PPFD tended to contain a relatively large number of photons below 600 nm and that with a higher PPFD tended to contain a relatively large number of photons above 700 nm (data not shown). Most likely, occasional reductions in PPFD by clouds that covered the sun enhanced the fraction of diffuse solar radiation from the sky in global solar radiation and the diffuse radiation was rich in light with a shorter waveband compared with direct radiation (Kume et al., 2018). Thus, both the PPFD and relative SPD of sunlight changed dynamically.

### Reproduction of the Time Course of Sunlight SPD With the LED-Artificial Sunlight Source System

Figure 4 also shows the time course of the PPFD of reproduced sunlight with the LASS system averaged over 12 replications (a blue line). The PPFD of reproduced sunlight agreed with that of actual sunlight except that it was lower than that of actual sunlight when the actual sunlight PPFD was greater than 1,200 μmol m^–2^ s^–1^ (Figure 4). Overall, the difference in PPFD between actual and reproduced sunlight at a given time was minor and considered to be acceptable.

**FIGURE 5:**
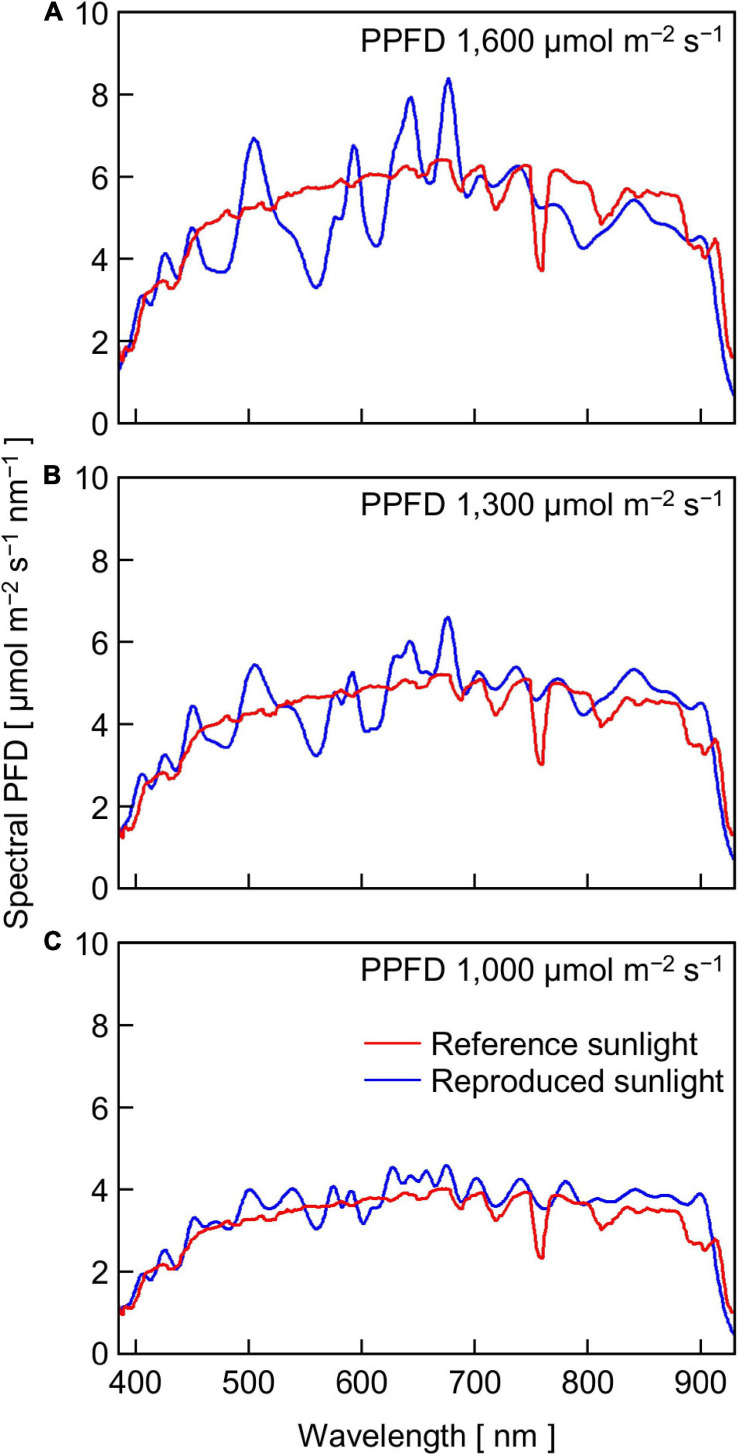
The SPDs of reference sunlight at PPFDs of 1,600 **(A)**, 1,300 **(B)**, and 1,000 **(C)** μmol m^–2^ s^–1^ and reproduced sunlight of which SPDs were approximated to those of the reference sunlight with the LASS system.

The relatively lower reproducibility of artificial sunlight PPFD in the high PPFD range was primarily due to the limited maximum output capacity of the LASS system, although it was reported that the LASS system could reproduce full irradiation of ground-level sunlight (Fujiwara et al., 2013). Specifically, there were two main reasons for the limitation generated in this experiment. One reason was that we did not use the re-approximation function in this study (see section “Materials and Methods”). Figure 5 shows the reference sunlight spectra with PPFDs of 1,600, 1,300, and 1,000 μmol m^–2^ s^–1^, as well as those of reproduced sunlight without the re-approximation function. The extent of approximation of the reproduced sunlight to the reference sunlight spectrum declined as the target PPFD increased: the coefficients of variation calculated at every 1 nm between 380 and 940 nm were 13.6, 15.0, and 18.4% for 1,000, 1,300, and 1,600 μmol m^–2^ s^–1^, respectively. The other reason was that the transparent polypropylene film (Propafilm C) covering the leaf chamber of the portable photosynthesis system significantly reduced the PPFD on the leaf surface. The spectral transmissivity of the film was approximately 85–90% between 380 and 940 nm and hardly dependent on wavelength (Meiwafosis Co., Ltd., personal communication), indicating that the film reduced SPDs within this range to a similar extent. However, the extent of sunlight SPD reproduction here must be the highest among those employed in previous experiments investigating the effects of fluctuating light on photosynthesis.

### Time Course of Leaf Gas Exchange Rates Under Reproduced Sunlight

**FIGURE 6:**
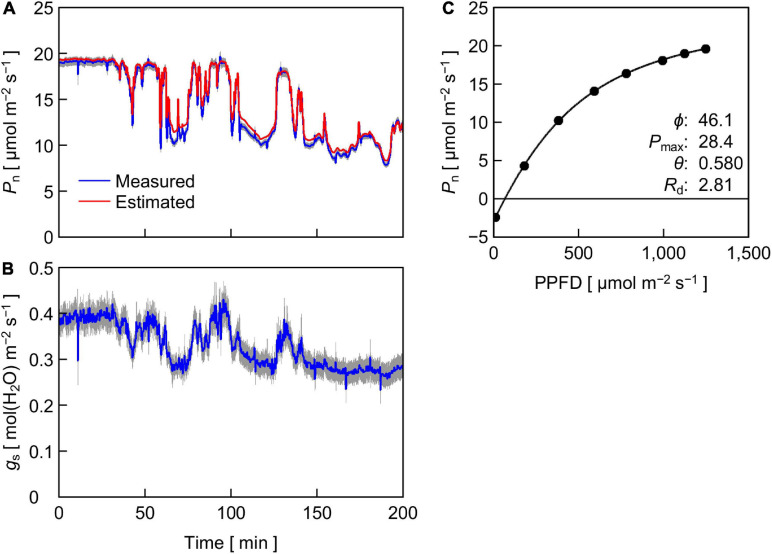
**(A,B)** Time course of *P*_n_ **(A)** and stomatal conductance *g*_s_ **(B)** in cucumber leaves measured under reproduced sunlight. For *P*_n_, values estimated from the steady-state PPFD-response curve of *P*_n_ **(C)** are also shown. The height of gray area at a given time represents the standard errors of the means for the measured values (*n* = 12). **(C)** Steady-state *P*_n_ in cucumber leaves in response to PPFD. Solid circles are the means of measured values (*n* = 12), and the line is a fitting curve with a nonrectangular hyperbolic function, of which parameter values are shown within the panel. Standard errors of the means are smaller than the diameter of the circles and are invisible.

Figure 6A shows the time course of *P*_n_ in cucumber leaves measured under reproduced sunlight and *P*_n_ estimated from a PPFD-response curve of steady-state *P*_n_ in leaves of the same plants (Figure 6C). The measured and estimated *P*_n_ agreed well except when the PPFD was 500 μmol m^–2^ s^–1^ or lower (see Figure 3), where the measured *P*_n_ was lower than the estimated *P*_n_. The time course of measured *g*_s_ (Figure 6B) resembled that of measured *P*_n_, while the response of *g*_s_ to changes in PPFD appeared to be delayed relative to that of *P*_n_. A slow response of *g*_s_ to a change in PPFD has been frequently reported (e.g., Lawson et al., 2012; Slattery et al., 2018). As a result, the amplitude of fluctuation appeared smaller in *g*_s_ than in *P*_n_.

We grew cucumber seedlings under phosphor-converted white LEDs, of which relative SPD (Figure 2) was quite different from that of the reproduced sunlight (Figure 5). One notable difference was the spectral PFD of far-red light: the white LED light contained a less proportion of far-red light than the reproduced sunlight. In leaves grown under light containing less far-red light, light is preferentially absorbed by photosystem II (PSII) compared with photosystem I (PSI) (“PSII-light”), and the ratio of the amount of PSII to that of PSI (PSII/PSI ratio) decreases to counteract the imbalance excitation (Chow et al., 1990a, b; Melis, 1991; Walters and Horton, 1994, 1995; Wagner et al., 2008; Hogewoning et al., 2012). One might suggest that the shift of the growth light of “PSII-light” to the *P*_n_ measurement light of “PSI-light” affected the response of *P*_n_ to the sunlight SPD fluctuations. However, Murakami et al. (2016) showed that cucumber leaves grown under phosphor-converted white LED light supplemented with and without far-red LED light did not show a significant difference in steady-state *P*_n_ measured under reproduced sunlight. This suggests that the effect of the shift from “PSII-light” during growth to “PSI-light” for *P*_n_ measurement in this study was also not significant.

### Relationship Between the Ratio of Measured *P*_n_ to Estimated *P*_n_ and PPFD or the Change in PPFD

**FIGURE 7:**
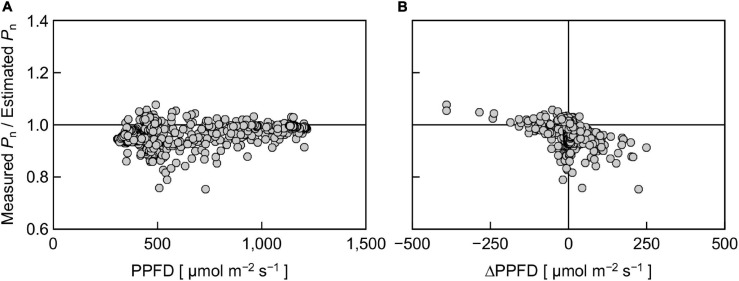
The ratio of measured *P*_n_ to estimated *P*_n_ in response to PPFD at a given moment **(A)** and the ratio in response to a change in PPFD for 15 s (ΔPPFD) **(B)**. Positive and negative ΔPPFD values represent increases and decreases in PPFD, respectively.

To further analyze the effect of reproduced sunlight PPFD on the difference between measured and estimated *P*_n_, the ratio of measured *P*_n_ to estimated *P*_n_ was plotted against PPFD (Figure 7A). Overall, a large part of the ratio was distributed below 1, indicating that the measured *P*_n_ was generally lower than the estimated *P*_n_. The ratio appeared to vary in an intermediate PPFD range of 400–700 μmol m^–2^ s^–1^ compared with lower and higher PPFD ranges. The linear regression was not statistically significant (*r*^2^ = 0.195). We summarized these data by averaging the measured and estimated *P*_n_, respectively, within every 200 μmol m^–2^ s^–1^ PPFD range between 300 and 1,300 μmol m^–2^ s^–1^ and computed the ratio (Table 1). The ratio was relatively low at low PPFDs; 0.95 and 0.94 for PPFD ranges of 300–500 and 500–700 μmol m^–2^ s^–1^, respectively. On the other hand, this value was slightly higher at high PPFDs; 0.98 and 0.99 for 900–1,100 and 1,100–1,300 μmol m^–2^ s^–1^, respectively. The overall ratio of measured *P*_n_ to estimated *P*_n_ between 300 and 1,300 μmol m^–2^ s^–1^ was 0.97, indicating that the reduction in *P*_n_ measured under reproduced sunlight compared with *P*_n_ estimated from the steady-state PPFD–*P*_n_ curve throughout the measurement was 3%.

**TABLE 1:** The ratio of mean measured *P*_n_ to mean estimated *P*_n_ calculated in different PPFD ranges.

**PPFD range (μmol m^–2^ s^–1^)**	**Measured *P*_n_ (μmol m^–2^ s^–1^)**	**Estimated *P*_n_ (μmol m^–2^ s^–1^)**	**Measured *P*_n_/estimated *P*_n_**
300–500	10.4	10.8	0.95
500–700	12.9	13.8	0.94
700–900	15.9	16.5	0.96
900–1,100	18.0	18.3	0.98
1,100–1,300	19.0	19.2	0.99
All (300–1,300)	14.3	14.7	0.97

This 3% reduction was significantly smaller than the 20–30% reduction reported in previous experimental (Vialet-Chabrand et al., 2017b) and simulation (Taylor and Long, 2017; Tanaka et al., 2019) studies but close to the 3–6% reduction on average reported by a more recent study employing comprehensive simulation over a wide range of diurnal PPFD fluctuations (Murakami and Jishi, 2021). There are several possible reasons for the difference between the values of calculated reduction. The first reason is the relative SPD. Reproducing both the PPFD and the relative SPD of sunlight could reduce the difference between the measured and estimated *P*_n_, compared with reproducing PPFD only. The second reason is the pattern of PPFD change. The difference between the measured and estimated *P*_n_ can depend on the pattern of PPFD change (Naumburg and Ellsworth, 2002). Murakami and Jishi (2021) also performed a simulation of diurnal courses of *P*_n_ under various PPFD fluctuation patterns of sunlight using a steady-state photosynthesis model and a dynamic photosynthesis model incorporating the response delay of *P*_n_ to an increase in PPFD. They showed that the difference in *P*_n_ calculated with the two models was largely dependent on the PPFD fluctuation pattern. The amplitudes of PPFD fluctuations in previous studies were ca. 100–2,000 μmol m^–2^ s^–1^ (Taylor and Long, 2017), 100–1,500 μmol m^–2^ s^–1^ (Vialet-Chabrand et al., 2017b), and 200–2,200 μmol m^–2^ s^–1^ (Tanaka et al., 2019), which are greater than those in the present study (400–1,300 μmol m^–2^ s^–1^). According to our data, the difference between the measured and estimated *P*_n_ tended to be high under low PPFD conditions (Figure 7A and Table 1). The levels and duration of low PPFDs in the PPFD fluctuating pattern, in relation to the shape of the PPFD-response curve of *P*_n_ in leaves considered, may be important to account for the difference between the measured and estimated *P*_n_. The third reason is the *P*_n_ measurement duration under fluctuating light. Vialet-Chabrand et al. (2017b) reported that when the overall PPFD level was high (mean: 460 μmol m^–2^ s^–1^), the extent of measured *P*_n_ reduction compared with the estimated *P*_n_ became greater, especially after 4–6 h after the measurement started. However, when the mean PPFD was low (230 μmol m^–2^ s^–1^), the reduction was apparent at the beginning of the measurement (Vialet-Chabrand et al., 2017b). The interactive effects of the fluctuating PPFD pattern and the timing at which the measured and estimated *P*_n_ started to significantly differ should be examined in detail in future work.

Finally, we evaluated the effect of PPFD change (ΔPPFD) on the ratio of measured *P*_n_ to estimated *P*_n_ (Figure 7B). There was a negative trend between the ratio and ΔPPFD; a large increase and decrease in PPFD tended to decrease and increase the ratio, respectively, although the linear regression was not statistically significant (*r*^2^ = 0.166). This trend may partly reflect the response delay of the portable photosynthesis system. In particular, the overvalued *P*_n_ when ΔPPFD was negative was likely due to the response delay, as the response of *P*_n_ to a decrease in PPFD was reportedly faster than that to an increase in PPFD (Bhuiyan and van Iersel, 2021). On the other hand, this trend suggests that *P*_n_ estimated using the steady-state PPFD-response curve (Figure 6C) tended to be particularly undervalued under the fluctuating SPD condition when the rate of PPFD increase was high. A similar result was reported by Bhuiyan and van Iersel (2021) that it took a longer time until *P*_n_ reached a steady state when the extent of PPFD increase was high.

## Conclusion

In this study, we reproduced a time course of sunlight SPD (both PPFD and relative SPD) using the LASS system. The *P*_n_ of cucumber leaves measured under the reproduced sunlight and that estimated from the steady-state PPFD–*P*_n_ curve of the same leaves were compared. The measured *P*_n_ tended to be lower than the estimated *P*_n_ under low PPFD conditions. The extent of measured *P*_n_ reduction compared with the estimated *P*_n_ averaged over all PPFD levels was 3%, which was smaller than the values of approximately 20–30% reported by previous studies (Taylor and Long, 2017; Vialet-Chabrand et al., 2017b; Tanaka et al., 2019). This finding suggests that the loss of integral net photosynthetic gain under fluctuating sunlight can vary among days with different fluctuation patterns or may be nonsignificant when fluctuations in both PPFD and relative SPD of sunlight are reproduced. More experimental observations of *P*_n_ under various patterns of reproduced fluctuating sunlight must be acquired and analyzed to discuss the quantitative importance of considering sunlight SPD fluctuations in leaf instantaneous photosynthesis.

## Data Availability Statement

The original contributions presented in the study are included in the article, further inquiries can be directed to the corresponding author.

## Author Contributions

RM conceived and designed the study and drafted the manuscript. HI acquired the data. HI and KF critically revised the manuscript. All authors analyzed the data and approved the final version.

## Conflict of Interest

The authors declare that the research was conducted in the absence of any commercial or financial relationships that could be construed as a potential conflict of interest.

## Abbreviations

DC: direct current
*g*_s_: stomatal conductance
LASS system: LED-artificial sunlight source system
LED: light-emitting diode
PFD: photon flux density
PPFD: photosynthetic PFD
SPD: spectral photon-flux-density distribution
*P*_n_: net photosynthetic rate.
